# Albumin level and risk of major bleeding in patients with atrial fibrillation on direct oral anticoagulants

**DOI:** 10.1093/ehjcvp/pvaf030

**Published:** 2025-04-24

**Authors:** Shun Sasaki, Daisuke Sakamoto, Yuki Matsuoka, Katsuki Okada, Akihiro Sunaga, Daisaku Nakatani, Hidetaka Kioka, Takashi Kanda, Hitoshi Minamiguchi, Ryuta Watanabe, Kouichi Nagashima, Yoshiharu Higuchi, Yasuo Okumura, Yohei Sotomi, Yasushi Sakata

**Affiliations:** Department of Cardiovascular Medicine, Osaka University Graduate School of Medicine, 2-2 Yamadaoka, Suita, Osaka 565-0871, Japan; Department of Cardiovascular Medicine, Osaka University Graduate School of Medicine, 2-2 Yamadaoka, Suita, Osaka 565-0871, Japan; Department of Cardiovascular Medicine, Osaka University Graduate School of Medicine, 2-2 Yamadaoka, Suita, Osaka 565-0871, Japan; Department of Cardiovascular Medicine, Osaka University Graduate School of Medicine, 2-2 Yamadaoka, Suita, Osaka 565-0871, Japan; Department of Medical Informatics, Osaka University Graduate School of Medicine, 2-2 Yamadaoka, Suita, Osaka 565-0871, Japan; Department of Cardiovascular Medicine, Osaka University Graduate School of Medicine, 2-2 Yamadaoka, Suita, Osaka 565-0871, Japan; Department of Cardiovascular Medicine, Osaka University Graduate School of Medicine, 2-2 Yamadaoka, Suita, Osaka 565-0871, Japan; Department of Cardiovascular Medicine, Osaka University Graduate School of Medicine, 2-2 Yamadaoka, Suita, Osaka 565-0871, Japan; Cardiovascular Division, Osaka Keisatsu Hospital, 2-6-40 Karasugatsuji, Tennoji-ku, Osaka 543-8922, Japan; Cardiovascular Division, Osaka Keisatsu Hospital, 2-6-40 Karasugatsuji, Tennoji-ku, Osaka 543-8922, Japan; Division of Cardiology, Department of Medicine, Nihon University School of Medicine, 30-1 Oyaguchi-kamicho, Itabashi-ku, Tokyo 173-8610, Japan; Division of Cardiology, Department of Medicine, Nihon University School of Medicine, 30-1 Oyaguchi-kamicho, Itabashi-ku, Tokyo 173-8610, Japan; Cardiovascular Division, Osaka Keisatsu Hospital, 2-6-40 Karasugatsuji, Tennoji-ku, Osaka 543-8922, Japan; Division of Cardiology, Department of Medicine, Nihon University School of Medicine, 30-1 Oyaguchi-kamicho, Itabashi-ku, Tokyo 173-8610, Japan; Department of Cardiovascular Medicine, Osaka University Graduate School of Medicine, 2-2 Yamadaoka, Suita, Osaka 565-0871, Japan; Department of Cardiovascular Medicine, Osaka University Graduate School of Medicine, 2-2 Yamadaoka, Suita, Osaka 565-0871, Japan

**Keywords:** Albumin, Atrial fibrillation, Anti-coagulation, DOAC, High bleeding risk, Protein binding

## Abstract

**Aims:**

The pharmacological effect of direct oral anticoagulants (DOACs) is influenced by binding status with albumin. This study aimed to assess the association between albumin levels and bleeding risk in atrial fibrillation (AF) patients treated with DOACs.

**Methods and results:**

We conducted DIRECT-Extend registry (*N* = 7512), a pooled database combining three large-scale observational study of AF patients treated with DOAC. The primary endpoint was major bleeding as defined by International Society on Thrombosis and Haemostasis criteria. Multivariable Cox hazard model was used to assess the impact of albumin level on major bleeding. Out of the overall cohort, 2523 patients [73 (IQR 66–80) years, 1620 (64.2%) males] with albumin data available at enrolment were analyzed in this study. Median follow-up duration was 532 days (IQR 94–1405 days). The entire cohort was divided into tertiles based on albumin levels (lower tertile: <3.7 g/dL, middle tertile: 3.7–4.1 g/dL, and higher tertile: ≥4.1 g/dL). The incidences of major bleeding increased as albumin levels decreased; 56 patients (6.8%), 81 patients (9.7%), and 113 patients (13.1%) in the higher, middle, and lower tertiles, respectively. (Log-rank test *P* < 0.0001). A lower albumin level was independently associated with a higher incidence of major bleeding (adjusted hazard ratio 0.61, 95% confidence interval 0.47–0.80, *P* < 0.01), which was consistently observed in all DOACs (*P*-value for interaction >0.05).

**Conclusion:**

A lower albumin level was independently associated with a higher bleeding risk in AF patients using DOACs. Careful attention should be paid to hypoalbuminemia when prescribing DOACs.

## Graphical Abstract

**Graphical Abstract:**
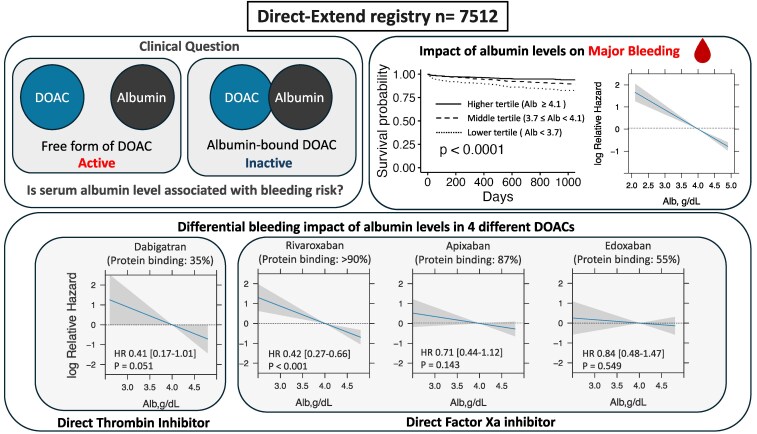

## Introduction

Patients with atrial fibrillation (AF) are recommended to consider anti-coagulation therapy to prevent stroke.^1–3^ Currently, direct oral anticoagulant (DOAC) is a first-line treatment for patients with AF. In general, DOAC binds to serum albumin at a certain level in the bloodstream and transitions between bound and unbound forms according to the dissociation equilibrium constant.^4,5^ The unbound free form of the drug is known to be responsible for its therapeutic effect.^6,7^ Therefore, the reduction of albumin will increase free drug concentrations, enhancing the drug's effect.^8^ However, hypoalbuminemia has not been well investigated as a bleeding risk factor for patients on DOAC. Hypoalbuminemia is not included in any bleeding risk scores (HAS-BLED score,^9^ ORBIT score^10^ and DOAC score^11^) or in the guidelines for bleeding risk assessment.^1–3^ Additionally, despite its role in drug metabolism, hypoalbuminemia is not included in the dosage adjustment criteria for all DOACs. This study aimed to investigate the association between albumin level and bleeding risk in AF patients on DOACs, using data from a real-world multi-centre observational study.

## Methods

### Study population

We established the DIRECT-Extend registry (UMIN000050585), a pooled database combining three large-scale observational study of non-valvular atrial fibrillation (NVAF) patients treated with anti-coagulation [DIRECT prospective registry (*N* = 2539), SAKURA-AF prospective registry (*N* = 3237), and Osaka University retrospective registry (*N* = 1736)]. Patients who visited or admitted at the study institutions (Osaka University Hospital, Osaka Keisatsu Hospital, and 72 facilities participating in SAKURA-AF registry) and were newly prescribed anticoagulants for NVAF were enrolled.

The inclusion and exclusion criteria for the 3 registries are summarized in Supplementary material online, *Table S1*. Briefly, in the DIRECT registry, all serial adult patients (aged ≥18 years) with NVAF at Osaka Keisatsu Hospital, who initiated DOAC (dabigatran, rivaroxaban, apixaban, or edoxaban) from June 2011 to November 2021, were prospectively enrolled.^12^ In the SAKURA-AF Registry, all adult patients (aged ≥20 years) with a diagnosis of NVAF, who were newly prescribed warfarin or DOACs for stroke prevention and had a follow-up duration of at least 1 year, were prospectively enrolled between September 2013 and December 2015. Participating institutions included two cardiovascular centres (Nihon University Itabashi Hospital and Nihon University Hospital), 13 affiliated or community hospitals, and 48 private clinics, all located mainly in the Tokyo metropolitan area.^13^ In the registry of Osaka University, we retrospectively collected clinical data of all adult NVAF patients who initiated DOAC therapy from May 2011 to November 2021 from electronic medical records. Baseline clinical characteristics were collected at the time of the first prescription of DOAC or warfarin. The DIRECT-Extend Registry complies with all the principles of the Declaration of Helsinki, and the study protocol was approved by the ethical committee of each institution.

### Albumin measurement

Serum albumin concentrations were measured using the bromocresol purple method, a widely used colorimetric assay. The most recent available data within 6 months prior to the enrolment date were used in this study. If multiple measurements were available, the data closest to enrolment was selected.

### The usage of DOAC

The medications were selected by physicians and were generally prescribed in accordance with the package insert. ‘Low-dose DOAC’ was defined as a composite of appropriate low-dose and under-dose. Appropriate low-dose refers to a reduced dosage given in compliance with the recommendations in the package insert. Patients with under-dose of DOAC indicate those who should have standard dose of DOAC but had a reduced dose at a physician’s discretion. Standard criteria of dose reduction for each DOAC were summarized in Supplementary material Appendix.

### Study design and endpoint

Study design is a post-hoc retrospective analysis of the pooled dataset of multiple registries. The present study aimed to assess the impact of albumin levels for the bleeding risk in NVAF patients using DOACs. The patients were divided into three tertiles based on their serum albumin levels.

Primary endpoint was major bleeding according to the International Society on Thrombosis and Haemostasis criteria which was defined as (i) fatal bleeding; and/or (ii) symptomatic bleeding in a critical area or organ (intracranial, intraspinal, intraocular, retroperitoneal, intra-articular or pericardial, or intramuscular with compartment syndrome); and/or (iii) bleeding causing a fall in haemoglobin level of ≥20 g/L (1.24 mmol/L), or leading to transfusion of ≥2 units of whole blood or red cells.^14^ Secondary endpoints were all-cause death, clinically relevant non-major bleeding defined according to the International Society on Thrombosis and Haemostasis criteria,^15^ any bleeding which is a composite of major bleeding and clinically relevant non-major bleeding, ischaemic stroke, hemorrhagic stroke, systemic embolism, and heart failure admission. Stroke was defined as a neurological deficit persisting ≥24 h attributed to an acute focal injury of the central nervous system by a vascular cause, including cerebral infarction, intracerebral haemorrhage, and subarachnoid haemorrhage. Systemic embolism was defined as an abrupt episode of arterial insufficiency associated with clinical or radiologic evidence of arterial occlusion in the absence of other likely mechanisms (e.g. atherosclerosis).^16^

### Statistical analysis

The statistical analysis was performed using R version 4.4.0. Categorical variables are expressed as counts (percentages) and compared with the χ^2^ test or Fisher’s exact test. Continuous variables are expressed as mean (SD) or median (interquartile range) and compared using Student *t* test or the Mann–Whitney U test as appropriate. Kaplan-Meier survival curves were constructed to estimate the cumulative incidence of major bleeding events across the three albumin tertiles, with differences assessed using the log-rank test. Because excluding missing data cases can cause bias in this analysis and loss of power for detecting a statistical difference, we performed random imputation using the ‘missForest’ method.^17^

Cox proportional hazards model was used to analyze albumin levels as a continuous variable while adjusting for multiple covariates and preserving statistical power. The proportional hazards assumption for serum albumin in relation to major bleeding was assessed using Schoenfeld residuals, yielding a *P*-value of 0.007, suggesting a violation of the proportional hazards assumption. Given this, we divided the follow-up period into two periods: within 30 days and beyond 30 days. Cox proportional hazards models were applied separately to each period to assess the association between albumin levels and major bleeding. For the model with a follow-up duration within 30 days, the test result was *P* = 0.14, while for the model with a follow-up duration of 30 days or more, the result was *P* = 0.12, suggesting no significant violation of the proportional hazards assumption for albumin in either models. Given this, we further examined the association between albumin levels and bleeding risk in both time periods and found consistent results between both time periods (details are described later). Although the violation of the proportional hazards assumption over the entire follow-up period necessitated a time-split analysis, the consistency of the findings across both periods indicated that the association between albumin levels and bleeding risk was robust. Therefore, despite this initial violation, we applied the Cox model to the analyses for the entire follow-up period as averaged estimates for the overall risk due to their ease of interpretation.

Given the importance of accurately modelling the association between albumin levels and major bleeding risk, we further examined whether a linear assumption was appropriate or if a more flexible functional form, such as a spline model, would provide a better fit. Both spline and linear models were considered for the analysis. For spline model, we evaluated with knots ranging from 3 to 7 using the Akaike Information Criterion (AIC). 3 knots resulted in the minimum AIC (3 knots: 3449.3; 4 knots: 3450.9; 5 knots: 3451.4; 6 knots: 3452.1; 7 knots: 3453.6). The likelihood ratio test comparing linear and spline models yielded a *P*-value of 0.63, indicating that the relationship between albumin levels and HR is adequately modelled by a linear term (see Supplementary material online, *Figure S1*). Therefore, we ultimately adopted the linear model in the Cox proportional hazards analysis. The covariates for Cox proportional hazards model were age, female sex, body mass index, diabetes mellitus, hypertension, history of bleeding, history of stroke, malignant neoplasm, haemoglobin levels, creatinine clearance, C-reactive protein, liver dysfunction, antiplatelet therapy, and NSAIDs use. These factors were determined by reference to the components of the HASBLED score,^9^ ORBIT score,^10^ DOAC score^11^ and previous our findings.^18^ The covariates for Cox proportional hazards model for stroke events (Ischaemic stroke, systemic embolism, haemorrhagic stroke) were components of CHA_2_DS_2_-VASc score.^19^ The covariates for heart failure admission were age, female, body mass index, diabetes mellitus, hypertension, vascular disease, history of heart failure, haemoglobin levels and creatinine clearance. These covariates were selected based on the clinical consensus. The covariates for all cause death were a combination of the components used for major bleeding and stroke. To individually evaluate the impact of albumin levels for the bleeding risk for four different DOACs, Cox hazard models was constructed respectively with the same covariates.

To evaluate the improvement in predictive performance for 1-year major bleeding with the addition of albumin, we constructed predictive models using a logistic regression model and compared the Area Under the Curve (AUC) of the receiver operating characteristic (ROC) curves for the original and modified risk scores (ORBIT, DOAC, and HAS-BLED with albumin included). The DeLong test was used to compare AUCs. Additionally, the Net Reclassification Improvement (NRI) and Integrated Discrimination Improvement (IDI) were calculated to assess the reclassification and discrimination performance, respectively.

## Results

### Study subjects

**Figure 1:**
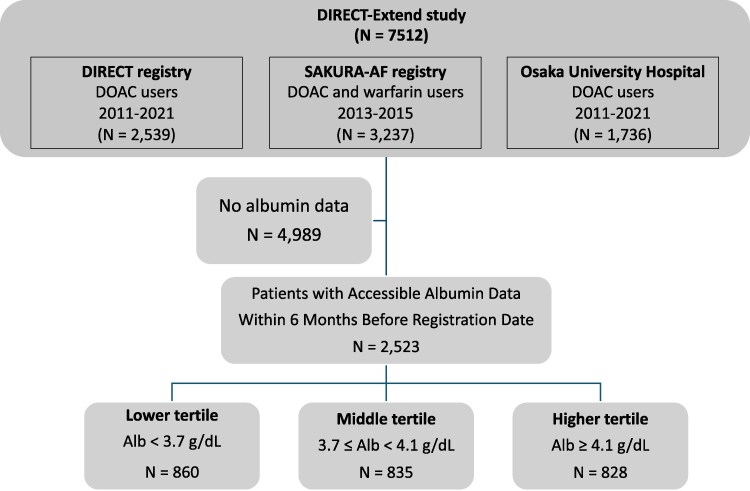
Study flowchart. The patients were divided into three tertiles based on their serum albumin levels: the first tertile consisted of patients with serum albumin levels lower than 3.7 g/dL (lower tertile, *N* = 860), the second tertile included those with serum albumin levels between 3.7 and 4.1 g/dL (middle tertile, *N* = 835), and the third tertile comprised patients with serum albumin levels of 4.1 g/dL or higher (higher tertile, *N* = 828).

A total of 7512 patients were enrolled in this pooled database. Out of the overall cohort, 2523 patients [73 (IQR 66–80) years, 1620 (64.2%) males] with albumin data available at enrolment were analyzed in this study. Patient characteristics and clinical outcomes in groups with and without albumin data are presented in Supplementary material online, *Table S2*. The patients were divided into three tertiles based on their serum albumin levels: the first tertile consisted of patients with serum albumin levels <3.7 g/dL (lower tertile, *N* = 860); the second tertile included those with serum albumin levels between 3.7 and 4.1 g/dL (middle tertile, *N* = 835); and the third tertile comprised patients with serum albumin levels of 4.1 g/dL or higher (higher tertile, *N* = 828) (*Figure 1*). The baseline characteristics of the three groups are shown in *Table 1* and Supplementary material online, *Tables S3*–S5.

**Table 1:** Patient characteristics

Variable	Lower tertile	Middle tertile	Higher tertile	*P*-value	Missing (%)
	Alb < 3.7 g/dL	3.7 ≤ Alb <4.1	Alb ≥ 4.1 g/dL		
	*N* = 860	*N* = 835	*N* = 828		
Age	77.0 [70.0, 83.0]	74.0 [67.0, 79.0]	68.0 [58.0, 75.0]	<0.001	0
Female sex	347 (40.3)	326 (39.0)	230 (27.8)	<0.001	0
Body weight, Kg	58.0 [49.0, 67.0]	60.8 [52.0, 70.0]	64.0 [55.0, 73.0]	<0.001	0.2
Body mass index, Kg/m^2^	22.8 [19.8, 26.3]	23.1 [20.6, 26.3]	23.6 [20.9, 26.2]	0.025	1.7
AF type				0.019	0
Paroxysmal	512 (59.5)	495 (59.3)	516 (62.3)		
Persistent or long persistent	307 (35.7)	314 (37.6)	296 (35.7)		
Unknown	41 (4.8)	26 (3.1)	16 (1.9)		
Hypertension	627 (72.9)	544 (65.1)	503 (60.7)	<0.001	0
Diabetes mellitus	253 (29.4)	197 (23.6)	214 (25.8)	0.023	0
Dyslipidemia	416 (48.4)	410 (49.1)	432 (52.2)	0.256	0
Coronary artery disease	148 (17.2)	112 (13.4)	86 (10.4)	<0.001	0
History of stroke	299 (34.8)	210 (25.1)	165 (19.9)	<0.001	0
History of heart failure	466 (54.2)	306 (36.6)	229 (27.7)	<0.001	0
History of bleeding	218 (25.4)	123 (14.7)	81 (9.8)	<0.001	0
Vascular disease	284 (33.0)	199 (23.8)	158 (19.1)	<0.001	0
Smoking				0.390	1.3
Never	410 (48.3)	427 (51.8)	398 (48.7)		
Past	333 (39.3)	312 (37.9)	334 (40.8)		
Current	105 (12.4)	85 (10.3)	86 (10.5)		
Liver dysfunction	274 (31.9)	167 (20.0)	150 (18.1)	<0.001	0
History of malignant neoplasm	281 (32.7)	232 (27.8)	157 (19.0)	<0.001	0
CHADS_2_ score	3.0 [2.0, 4.0]	2.0 [1.0, 3.0]	2.0 [1.0, 3.0]	<0.001	0
CHA_2_DS_2_-VASc score	4.0 [3.0, 6.0]	4.0 [2.0, 5.0]	3.0 [1.0, 4.0]	<0.001	0
ORBIT score	3.0 [2.0, 4.0]	2.0 [1.0, 3.0]	1.0 [0.0, 2.0]	<0.001	0.3
DOAC score	8.0 [6.0, 10.0]	6.0 [4.0, 8.0]	4.0 [2.0, 7.0]	<0.001	2
HAS-BLED score	3.0 [2.0, 4.0]	3.0 [2.0, 4.0]	2.0 [1.0, 3.0]	<0.001	0.2

Data with listwise deletion are expressed as median [interquartile range] or number (percentage).

Abbreviations: Alb, Albumin; AF, Atrial fibrillation; DOAC, Direct oral anticoagulant.

### Impact of albumin levels on clinical outcomes

**Figure 2:**
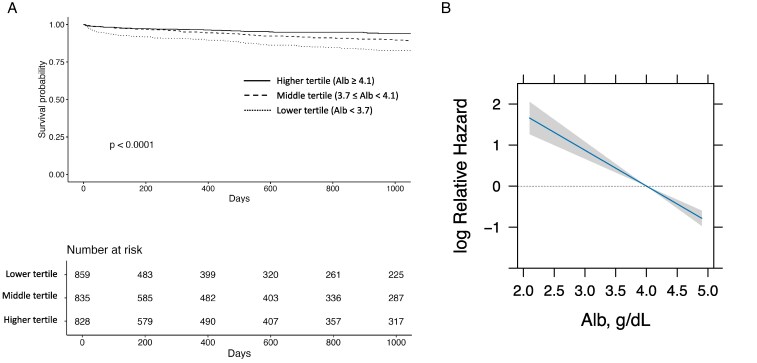
A lower albumin level is associated with a bleeding risk for AF patients using DOACs. (*A*) The Kaplan-Meier survival curves illustrate the survival probability over time stratified by albumin tertiles. The tertiles are categorized as Higher tertile (solid line), Middle tertile (dashed line), and Lower tertile (dotted line). (*B*) This figure illustrates the relationship between albumin levels (Alb, g/dL) and the relative hazard ratios. The impact of albumin levels was analyzed using a Cox proportional hazards model with a linear term for albumin. The covariates for Cox proportional hazards model were age, female sex, body mass index, diabetes mellitus, hypertension, history of bleeding, history of stroke, malignant neoplasm, haemoglobin levels, creatinine clearance, C-reactive protein, liver dysfunction, antiplatelet therapy, and NSAIDs use. The reference level of albumin was set at 4.0 g/dL. The solid lines represent the estimated hazard ratios, while the shaded grey areas indicate the 95% CIs. Relative hazard ratio is presented on a log^e^ (natural logarithm) scale. Alb, albumin; HR, hazard ratio.

Median follow-up duration was 532 days (IQR 94–1405 days). The incidences of major bleeding increased as albumin levels decreased; 56 patients (6.8%), 81 patients (9.7%), and 113 patients (13.1%) in the higher, middle, and lower tertiles, respectively. (Log-rank test *P* < 0.0001) (*Figure 2A*).

As described in the method section, we applied the Cox model to the entire follow-up period to provide averaged estimates for overall risk and present these results as primary findings. The number of clinical events, 100-person-year event rates, results of the multivariable Cox hazard model for the primary endpoint, and secondary endpoints are shown in *Table 2*. In the multivariable Cox proportional hazards model, higher serum albumin levels were significantly associated with a reduced risk of several adverse outcomes. Specifically, for each 1.0 g/dL increase in albumin, the risk of the primary endpoint (major bleeding) decreased {Hazard ratio (HR) [95% confidence interval (CI)] 0.61 (0.47–0.80), *P* < 0.01} (*Figure 2B*), as did the risks of clinically relevant non-major bleeding [HR 0.83 (0.73–0.95), *P* < 0.01] and any bleeding [HR 0.85 (0.74–0.98), *P* = 0.02]. Additionally, higher albumin levels were significantly associated with a lower risk of haemorrhagic stroke [HR 0.47 (0.26–0.86), *P* = 0.01] and all-cause mortality [HR 0.45 (0.35–0.57), *P* < 0.01]. In contrast, no significant associations were observed between albumin levels and the risks of ischaemic stroke [HR 0.88 (0.53–1.46), *P* = 0.62], systemic embolism [HR 0.67 (0.39–1.15), *P* = 0.15], or heart failure admission [HR 0.88 (0.70–1.12), *P* = 0.30].

**Table 2:** Clinical outcomes

Outcome	Number of events	100-person-year event rates				Cox proportional hazard model			
						Univariable analysis		Multivariable analysis	
		Lower tertile	Middle tertile	Higher tertile	*P*-value	HR [95% CI]	*P*-value	HR [95% CI]	*P*-value
		Alb < 3.7 g/dL	3.7 ≤ Alb <4.1	Alb ≥ 4.1 g/dL		per Alb 1.0 g/dL increase		per Alb 1.0 g/dL increase	
Major bleeding	250 (9.9%)	6.69	3.90	2.49	< 0.01	0.42 [0.34–0.51]	<0.01	0.61 [0.47–0.80]	< 0.01
Clinically relevant non-major bleeding	1085 (43.0%)	22.32	18.14	14.69	< 0.01	0.70 [0.63–0.78]	<0.01	0.83 [0.73–0.95]	< 0.01
Any bleeding	1148 (45.5%)	24.22	18.91	15.36	< 0.01	0.67 [0.60–0.74]	<0.01	0.85 [0.74–0.98]	0.02
Ischaemic stroke	62 (2.5%)	1.24	1.35	0.58	0.03	0.68 [0.43–1.08]	0.10	0.88 [0.53–1.46]	0.62
Systemic embolism	48 (1.9%).	1.13	1.06	0.31	< 0.01	0.51 [0.31–0.83]	< 0.01	0.67 [0.39–1.15]	0.15
Haemorrhagic stroke	35 (1.4%)	1.01	0.58	0.27	< 0.01	0.42 [0.25–0.73]	< 0.01	0.47 [0.26–0.86]	0.01
Heart failure admission	256 (10.2%)	6.34	4.04	2.88	< 0.01	0.56 [0.45–0.70]	< 0.01	0.88 [0.70–1.12]	0.30
All cause death	202 (8.0%)	6.51	2.74	1.55	< 0.01	0.33 [0.27–0.41]	<0.01	0.45 [0.35–0.57]	< 0.01

Cox proportional hazards regression analysis for bleeding events was performed including several covariates such as age, female sex, body mass index, diabetes mellitus, hypertension, history of bleeding, history of stroke, malignant neoplasm, haemoglobin levels, creatinine clearance, C-reactive protein, liver dysfunction, antiplatelet therapy, and NSAIDs use. These factors were determined by reference to the components of the HASBLED score,^9^ ORBIT score,^10^ DOAC score^11^ and previous our findings.^18^ The covariates for Cox proportional hazards model for stroke events (Ischaemic stroke, systemic embolism, haemorrhagic stroke) were components of CHA_2_DS_2_-VASc score.^19^ The covariates for heart failure admission were age, female, body mass index, diabetes mellitus, hypertension, vascular disease, history of heart failure, haemoglobin levels and creatinine clearance. These covariates were selected based on the clinical consensus. The covariates for all cause death were a combination of the components used for major bleeding and stroke.

Abbreviations: Alb, Albumin; HR, Hazard Ratio.

### Impact of albumin levels on major bleeding: a time-dependent cox regression analysis divided at 30 days

The Cox hazard model applied to the analyses for the primary endpoint in the entire follow-up period indicated the violation of the proportional hazards assumption (Schoenfeld residuals, *P* = 0.007), which necessitated a time-split analysis into two phases: within 30 days and beyond 30 days. Within the first 30 days, a lower albumin level was associated with a higher bleeding risk [HR (95% CI) 0.39 (0.23–0.64), *P* < 0.001]. This association remained significant beyond 30 days [HR 0.73 (0.54–1.00), *P* = 0.048], suggesting that a lower albumin level was consistently associated with a higher risk of major bleeding (see Supplementary material online, *Table S6*).

### Subgroup analysis

In the subgroup analysis using the Cox proportional hazards model stratified by various factors, the impact of albumin levels on hazard ratios was generally consistent across all subgroups (see Supplementary material online, *Figure S2*). In the subgroup analysis using Cox proportional hazards model stratified by albumin tertiles, low-dose DOAC was not significantly associated with the lower HR in any group [HR: 0.70 (0.44–1.12), *P* = 0.14 for the lower tertile; HR: 0.92 (0.55–1.54), *P* = 0.75 for the middle tertile; HR: 0.77 (0.41–1.43), *P* = 0.40 for the higher tertile] (see Supplementary material online, *Table S7*).

### Incremental value of albumin levels in predictive performance

**Figure 3:**
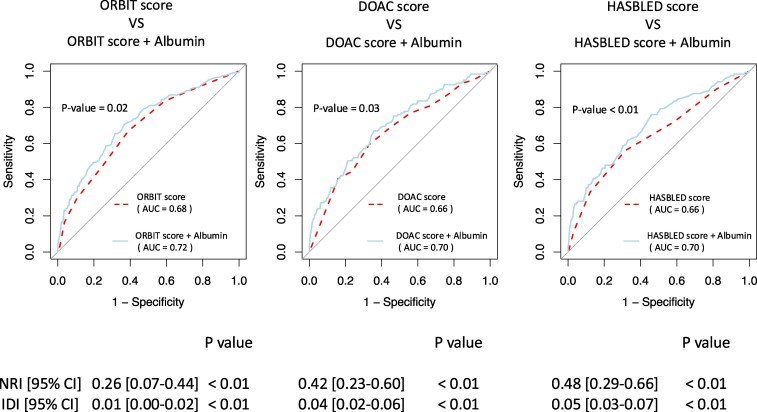
Improvement in predictive performance of bleeding risk scores for 1-year major bleeding with the inclusion of albumin. This figure shows the ROC curves for the ORBIT, DOAC, and HAS-BLED scores for 1-year major bleeding, comparing their predictive accuracy alone (dashed lines) vs. when combined with albumin (solid lines). IDI, Integrated discrimination improvement; NRI, Net reclassification improvement.

The inclusion of albumin levels significantly improved the predictive performance of the ORBIT, DOAC, and HAS-BLED scores for 1-year major bleeding. The inclusion of albumin improved the AUC for the ORBIT score from 0.68 to 0.72 (*P* = 0.02), with an NRI of 0.26 (95% CI: 0.07–0.44, *P* < 0.01) and an IDI of 0.01 (95% CI: 0.00–0.02, *P* < 0.01). Similarly, the DOAC score's AUC increased from 0.66 to 0.70 (*P* = 0.03), with an NRI of 0.42 (95% CI: 0.23–0.60, *P* < 0.01) and an IDI of 0.04 (95% CI: 0.02–0.06, *P* < 0.01). For the HAS-BLED score, the AUC improved from 0.64 to 0.70 (*P* < 0.01), with an NRI of 0.48 (95% CI: 0.29–0.66, *P* < 0.01) and an IDI of 0.05 (95% CI: 0.03–0.07, *P* < 0.01) (*Figure 3*).

### Impact of albumin levels on bleeding risk in 4 different DOACs

**Figure 4:**
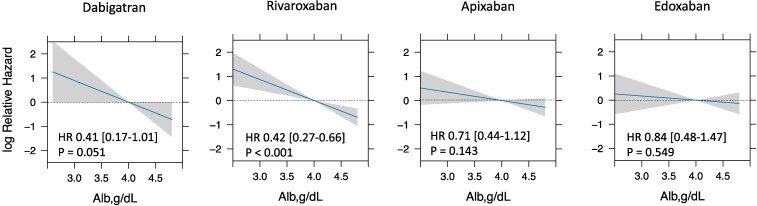
Effect of albumin levels on major bleeding in four different DOACs. This figure illustrates the relationship between albumin levels (Alb, g/dL) and the relative hazard ratios for four different DOACs. The impact of albumin levels on bleeding risk for each DOAC was analyzed using a Cox proportional hazards model with a linear term for albumin. The covariates for Cox proportional hazards model were age, female sex, body mass index, diabetes mellitus, hypertension, history of bleeding, history of stroke, malignant neoplasm, haemoglobin levels, creatinine clearance, C-reactive protein, liver dysfunction, antiplatelet therapy, and NSAIDs use. The reference level of albumin was set at 4.0 g/dL. The solid lines represent the estimated hazard ratios, while the shaded grey areas indicate the 95% CIs. Relative hazard ratio is presented on a log^e^ (natural logarithm) scale. Alb, albumin; HR, hazard ratio; DOAC, direct oral anticoagulant.

The baseline characteristics of the patients stratified by type of DOAC are shown in Supplementary material online, *Tables S8*–S10. The impact of albumin levels for major bleeding for four different DOACs using a multivariable Cox hazard model is shown in *Figure 4*. While hazards for major bleeding numerically increased as albumin levels decreased in all four different DOACs, only rivaroxaban demonstrated a statistically significant association between albumin levels and bleeding risk [HR: 0.42 (0.27–0.66), *P* < 0.01]. However, the *P*-value for interaction between albumin levels and four different DOACs, using dabigatran as a reference, was not statistically significant (rivaroxaban: *P* = 0.43, apixaban: *P* = 0.27, edoxaban: *P* = 0.78).

## Discussion

The main findings of this study are as follows: (i) a lower albumin level was independently associated with a higher risk of major bleeding in AF patients using DOACs; and (ii) the influence of albumin levels on bleeding risk was consistent among different DOACs. To the best of our knowledge, this is the first large-scale study to demonstrate that a lower albumin level is a bleeding risk factor of AF patients on any DOACs.

### A lower albumin level is a bleeding risk factor for AF patients using DOACs

Hypoalbuminemia has not previously been included as a risk factor in any guidelines or any bleeding risk scores because it was not recognized as a bleeding risk in AF patients using DOACs. However, this study has revealed that a lower albumin level is independently associated with a higher bleeding risk, separate from other known risk factors. While a previous study has demonstrated an association between lower albumin levels and a higher bleeding risk in patients treated with rivaroxaban^20^ (single-centre study, *N* = 368), our study extends this finding by showing similar results in a broader cohort of patients using various DOACs. This comprehensive analysis highlights the novelty of our research, offering a more generalisable insight into the relationship between a lower albumin level and a higher bleeding risk. Dynamic equilibrium depends on various factors, including the type of drug, distribution, metabolism, excretion, and albumin levels.^4^ Low albumin levels can result in elevated unbound DOACs and an increased bleeding risk.

In our analysis, although a lower albumin level was associated with an increased bleeding risk, there was no corresponding reduction in the risk of ischaemic stroke or systemic embolism. Hypoalbuminemia can alter the pharmacokinetics of DOACs, potentially increasing their free (active) drug concentration and enhancing their anticoagulant effect. However, this effect may be limited in terms of stroke prevention, as ischaemic stroke can result not only from cardiac embolism but also from other causes such as atherosclerosis, which DOACs may not fully prevent. Given this, the antithrombotic effect in patients with hypoalbuminemia may reach a plateau, whereas the bleeding risk continues to increase. These findings suggest that the current DOAC dosage may be excessive in patients with hypoalbuminemia. Considering these factors, dose reduction of DOACs in patients with hypoalbuminemia might be beneficial. In this study, we examined this hypothesis; however, the risk reduction with low-dose DOACs did not reach statistical significance, although the point estimates indicated a trend towards risk reduction. Further pharmacological investigations and well-powered prospective randomized controlled trials are needed to validate these findings.

### Impact of albumin levels on major bleeding in different DOACs

Albumin interacts with DOAC and is involved in their metabolism.^5^ The pharmacokinetic characteristics of DOAC are summarized in Supplementary material online, *Table S11*. The plasma protein binding percentages for different anticoagulants are as follows: dabigatran (35%), rivaroxaban (>90%), apixaban (87%), and edoxaban (55%).^5^ Previous studies from a single-centre cohort reported that a low albumin level was associated with bleeding risk in patients receiving rivaroxaban or warfarin, both of which exhibit high protein binding.^20–22^ In our study, while hazards for major bleeding numerically increased as albumin levels decreased in all 4 different DOACs, only rivaroxaban demonstrated a statistically significant association between albumin levels and bleeding risk. This is in line with the previous reports.^20–22^ Apixaban, despite its relatively high protein binding ratio, was not significantly affected by albumin levels. One possible explanation is that its twice-daily dosing may help stabilize plasma drug concentrations, preventing excessive fluctuations in free drug levels caused by low albumin. Since the increase in free DOAC concentration after administration is expected to be similar between apixaban and rivaroxaban, the twice-daily regimen of apixaban may distribute this effect more evenly throughout the day, thereby reducing its overall impact on bleeding risk.^23,24^ As theoretically expected, edoxaban, which has a relatively low protein binding percentage, was not affected by albumin levels. These findings may suggest that the impact of albumin levels on bleeding risk differs according to protein binding rates as for Xa inhibitors. On the other hand, unexpectedly, albumin levels had a relatively strong impact on bleeding risk in dabigatran despite its low protein binding ratio. This finding may be linked to dabigatran’s unique mechanism as a direct thrombin inhibitor, along with the potential for tartaric acid in dabigatran etexilate to induce direct mucosal injury.^25^ However, precise mechanisms remain unclear. This study demonstrated numerically differential impact of albumin levels among the different DOACs, although the statistical analysis indicated its consistent influence. Further research is required to examine the differential influence of protein binding rates on the association between albumin levels and bleeding risk.

### Limitation

Several limitations must be acknowledged. Firstly, this study is a multicenter East Asian AF registry, which restricts the generalizability of the findings to other racial groups. However, the dose criteria for Asian and non-Asian populations do not differ significantly, and the pharmacological effects of DOACs would remain similar across ethnic groups. Therefore, albumin may also serve as a potential risk factor for bleeding in DOAC-treated patients in non-Asian populations. Secondly, the small sample size, particularly in the subgroup analysis stratified by type of DOAC, may have resulted in a type II error. Consequently, the results should be interpreted with caution. Thirdly, clinical events were not adjudicated by a clinical events committee, and event classification solely relied on the available clinical records. Forth, our study is subject to selection bias due to the inclusion of only the population with available albumin data, which may affect the generalizability of our findings. Fifth, as this is a retrospective study, residual confounding and unmeasured variables cannot be completely ruled out. Sixth, this study demonstrated that a lower albumin level was independently associated with a higher major bleeding risk. However, due to the absence of data on blood drug concentrations of DOACs, it remains unclear whether low albumin levels truly led to increased concentrations of the unbound form of DOACs in this population. Seventh, we were unable to stratify analyses by the cause of death due to the lack of available data. This limitation prevents us from assessing whether the relationship between albumin levels and bleeding risk varies by mortality aetiology. Eighth, we did not collect data on bleeding events defined by other classifications such as GUSTO, TIMI, or BARC, which does not allow us to assess the relationship between albumin levels and bleeding risk using these criteria. Lastly, we cannot exclude the potential influence of unknown factors that may affect both albumin levels and bleeding risk. Further basic research is necessary to elucidate the specific mechanisms by which a lower albumin level contributes to a higher bleeding risk.

## Conclusions

A lower albumin level was independently associated with a higher bleeding risk in AF patients using DOACs. Careful attention should be paid to hypoalbuminemia when prescribing DOACs.

## Supplementary Material

pvaf030_Supplementary_Data

## Data Availability

Our study data will not be made available to other researchers for purposes of reproducing the results because of institutional review board restrictions.
