# Supplementary material for: Albumin level and risk of major bleeding in patients with atrial fibrillation on direct oral anticoagulants
Source: Eur Heart J Cardiovasc Pharmacother. 2025 Apr 24;11(5):422–32. doi: 10.1093/ehjcvp/pvaf030 (PMC12343015; doi:10.1093/ehjcvp/pvaf030)
Supplement: pvaf030_Supplementary_Data [file pvaf030_supplementary_data.docx]

**Supplementary data**

**Albumin Level and Risk of Major Bleeding in Patients with Atrial fibrillation on Direct Oral Anticoagulants: Insights from the DIRECT-Extend registry**

Method. Dosing criteria

Supplemental Figure 1. Linear and spline models for albumin levels to hazard ratios for the primary endpoint

Supplemental Figure 2. Impact of albumin levels on major bleeding: Subgroup analysis using Cox proportional hazards regression model

Supplemental Table 1. Impact of albumin levels on major bleeding: A time-dependent Cox regression analysis divided at 30 days

Supplemental Table 2. The inclusion and exclusion criteria for the three registries.

Supplemental Table 3. Medications

Supplemental Table 4. Laboratory data

Supplemental Table 5. Echocardiographic data

Supplemental Table 6. Patient characteristics and clinical outcome with and without albumin data of DIRECT-Extend study

Supplemental Table 7. Impact of low dose DOAC on major bleeding in albumin tertiles

Supplemental Table 8. Patient characteristics stratified by DOACs

Supplemental Table 9. Medications stratified by DOACs

Supplemental Table 10. Laboratory data stratified by DOACs

Supplemental Table 11. Pharmacokinetic characteristics of DOACs

**Method**

**Dosing Criteria**

The appropriate DOAC dosing is defined as follows, according to which DOAC agent is prescribed:

**Japan**

Dabigatran

- 150 mg twice daily is standard dose.
- Age ≥70 years, or CrCl 30 to 50 mL/min, or patient receiving concomitant P-gp inhibitors, or history of gastrointestinal bleeding, then consider reducing dabigatran to 110 mg twice daily.
- CrCl <30 mL/min: not recommended.

Rivaroxaban

- 20 mg once daily is standard dose.
- CrCl 15 to 49 mL/min: 15 mg once daily.
- CrCl <15 mL/min: Avoid use.

Apixaban

- 5 mg twice daily is standard dose.
- Patients with at least 2 of the following characteristics: Age ≥80 years, body weight ≤60 kg, or serum creatinine ≥1.5 mg/dL, then reduce dose to 2.5 mg twice daily.
- CrCl <15 mL/min: Avoid use.

Edoxaban

- 60 mg once daily is standard dose.
- Body weight ≤60 kg or CrCl 15 to 49 mL/min or patient receiving concomitant P-gp inhibitors: 30 mg once daily.
- CrCl <15 mL/min: Avoid use.

**United States**

Dabigatran

- 150 mg twice daily is standard dose.
- CrCl 30 to 50 mL/min: No dosage adjustment necessary unless patient receiving concomitant P-gp inhibitors, then consider reducing dabigatran to 75 mg twice daily.
- CrCl 15 to 30 mL/min: 75 mg twice daily unless patient receiving concomitant P-gp inhibitors, then avoid concurrent use.
- CrCl <15 mL/min: not recommended.

Rivaroxaban

- 20 mg once daily is standard dose.
- CrCl 15 to 50 mL/min: 15 mg once daily.
- CrCl <15 mL/min: Avoid use.

Apixaban

- 5 mg twice daily is standard dose.
- Patients with at least 2 of the following characteristics: Age ≥80 years, body weight ≤60 kg, or serum creatinine ≥1.5 mg/dL, then reduce dose to 2.5 mg twice daily.
- CrCl <15 mL/min: Avoid use.

Edoxaban

- 60 mg once daily is standard dose.
- CrCl 15 to 50 mL/min: 30 mg once daily.
- CrCl <15 mL/min or CrCl > 95 ml/min: Avoid use.
- Patient receiving concomitant rifampin: Avoid use.

**Europe**

Dabigatran

- 150 mg twice daily is standard dose.
- Age ≥80 years or patient receiving concomitant P-gp inhibitors, then consider reducing dabigatran to 110 mg twice daily.
- Patients with at least 2 of the following characteristics: Age 75–79 years, CrCl 30–50 mL/min, HAS-BLED ≥ 3, amiodarone use, platelet aggregation inhibitors use, or body weight ≤ 60 kg, then reduce dose to 110 mg twice daily.
- CrCl <30 mL/min: not recommended.

Rivaroxaban

- 20 mg once daily is standard dose.
- CrCl 15 to 49 mL/min: 15 mg once daily.
- Patients with at least 2 of the following characteristics: age ≥ 75 years, HAS-BLED ≥ 3, amiodarone use, platelet aggregation inhibitors use, or body weight ≤ 60 kg: 15 mg once daily.
- CrCl <15 mL/min: Avoid use.

Apixaban

- 5 mg twice daily is standard dose.
- Patients with at least 2 of the following characteristics: Age ≥80 years, body weight ≤60 kg, or serum creatinine ≥1.5 mg/dL, then reduce dose to 2.5 mg twice daily.
- Patients with at least 2 of the following characteristics: age ≥ 75 years, HAS-BLED ≥ 3, amiodarone use, platelet aggregation inhibitors use, or diltiazem use, then reduce dose to 2.5 mg twice daily.
- CrCl 15 to 29 mL/min: 2.5 mg twice daily.
- CrCl <15 mL/min: Avoid use.

Edoxaban

- 60 mg once daily is standard dose.
- Body weight ≤60 kg or CrCl 15 to 49 mL/min: 30 mg once daily.
- Patient receiving concomitant strong p-glycoprotein inhibitors, including cyclosporine, dronedarone, erythromycin, or ketoconazole, then reduce dose to 30 mg once daily.
- Patients with at least 2 of the following characteristics: age ≥ 75 years, HAS-BLED ≥ 3, amiodarone use, amiodarone or verapamil use, platelet aggregation inhibitors use then reduce dose to 30 mg once daily
- CrCl <15 mL/min: Avoid use.

**Supplemental Figure 1**

**Linear and spline models for albumin levels to hazard ratios for the primary endpoint**

**
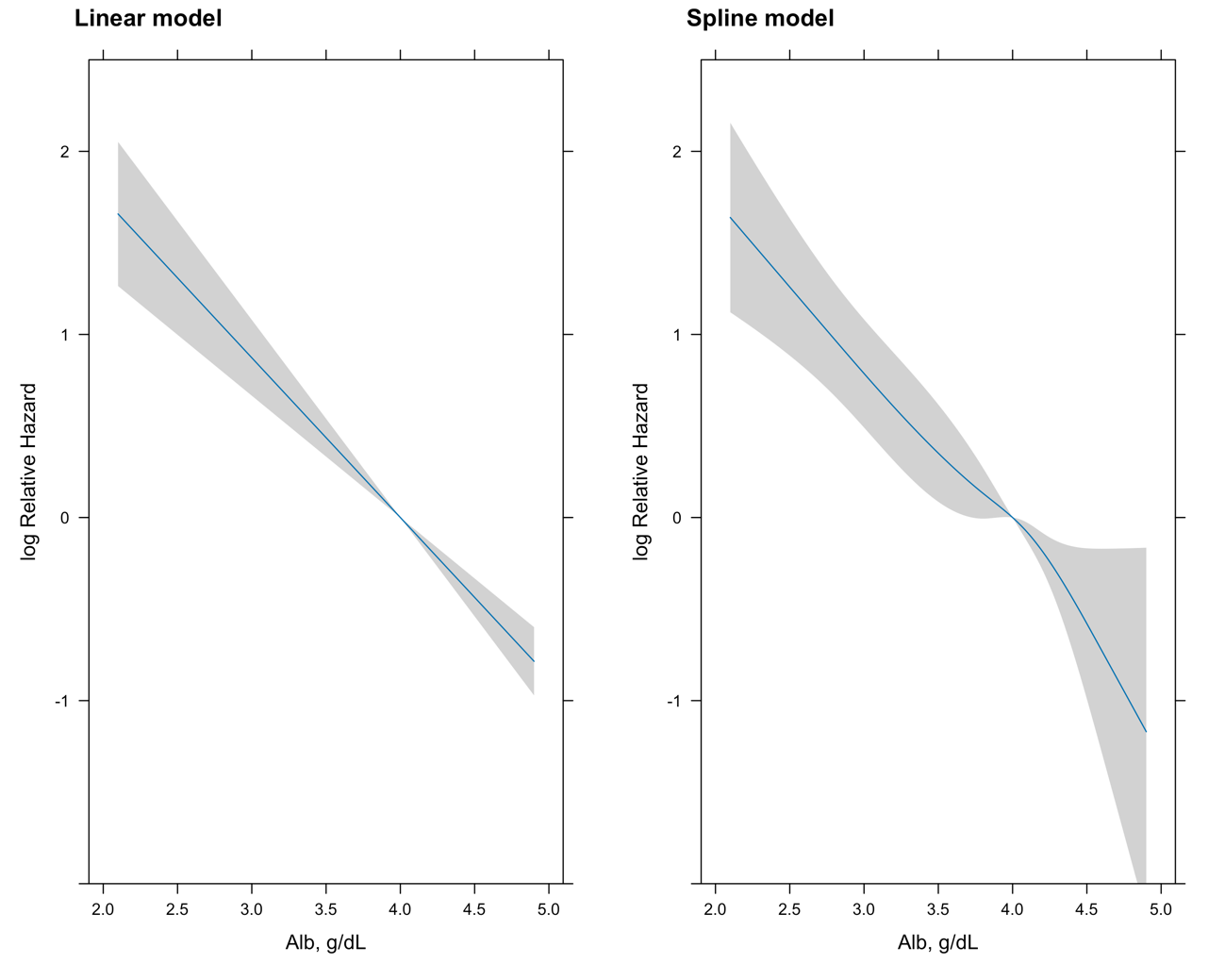
**

Both linear and spline models were considered for the analysis. For spline model, we evaluated with knots ranging from 3 to 7 using the Akaike Information Criterion (AIC). 3 knots resulted in the minimum AIC (3 knots: 3449.3; 4 knots: 3450.9; 5 knots:3451.4; 6 knots: 3452.1; 7 knots: 3453.6). The likelihood ratio test comparing linear and spline models yielded a p-value of 0.63, indicating that the relationship between albumin levels and HR is adequately modeled by a linear term. The covariates for Cox proportional hazards model were age, female sex, body mass index, diabetes mellitus, hypertension, history of bleeding, history of stroke, malignant neoplasm, hemoglobin levels, creatinine clearance, C-reactive protein, liver dysfunction, antiplatelet therapy, and NSAIDs use.

Abbreviations: Alb, albumin.

**Supplemental Figure 2**

**Impact of albumin levels on major bleeding: Subgroup analysis using Cox proportional hazards regression model**

This forest plot illustrates the hazard ratios (HRs) and 95% confidence intervals (CIs) for major bleeding across various subgroups, evaluating the association between albumin levels and bleeding risk. P-values less than 0.003125 were considered statistically significant (Bonferroni correction). A logarithmic scale was used for hazard ratios. Abbreviations: CKD, chronic kidney disease; NSAIDs, Non-steroidal anti-inflammatory drugs; DOAC, Direct oral anticoagulant.

*Younger patients were defined as those aged <75 years, and elderly patients as those aged ≥75 years.

†Obesity was defined as BMI ≥25 kg/m² according to the Japanese criteria.

‡Anemia (-) and Anemia (+) were classified according to the WHO criteria, defined as hemoglobin levels below 13.0 g/dL for men and 12.0 g/dL for women (WHO, 2011).

**CKD was defined as an eGFR of less than 60 mL/min/1.73 m², and non-CKD as an eGFR of 60 mL/min/1.73 m² or higher.

**Supplemental Table 1**

***Impact of albumin levels on major bleeding: A time-dependent Cox regression analysis divided at 30 days***

| **Variable** | **Within 30 days** | | **Beyond 30 days** | |  |
| --- | --- | --- | --- | --- | --- |
|  | **N = 2522** |  | **N = 2122** |  | |
|  | **HR [95% CI]** | **P value** | **HR [95% CI]** | **P value** | |
| **Albumin, g/dL** | 0.39 [0.23-0.64] | <0.001 | 0.73 [0.54-1.00] | 0.048 | |

The covariates for Cox proportional hazards model were age, female sex, body mass index, diabetes mellitus, hypertension, history of bleeding, history of stroke, malignant neoplasm, hemoglobin levels, creatinine clearance, C-reactive protein, liver dysfunction, antiplatelet therapy, and NSAIDs use.

Abbreviations: NSAIDs, Non-steroidal anti-inflammatory drugs.

**Supplemental Table 2. The inclusion and exclusion criteria for the three registries.**

|  | **DIRECT registry** | **SAKURA-AF registry** | **The registry of Osaka University** |
| --- | --- | --- | --- |
| **Inclusion criteria** | - Aged ≥18 years. - A diagnosis of non-valvular atrial fibrillation. - All consecutive patients who initiated DOAC therapy between June 2011 and November 2021 in the Osaka Keisatsu hospital. | - Aged ≥20 years. - A diagnosis of non-valvular atrial fibrillation. - Prescribed warfarin or DOACs for stroke prevention. - Follow-up duration ≥ 1 year. | - Aged ≥18 years. - A diagnosis of non-valvular atrial fibrillation. - All consecutive patients who initiated DOAC therapy between May 2011 and November 2021 in the Osaka University hospital. |
| **Exclusion criteria** | - Patients who were prescribed DOACs for the treatment of deep vein thrombosis. | - Patients with rheumatic mitral valve disease, history of prosthetic valve replacements, active infective endocarditis. - Patients who failed to provide written informed consent. | - Patients who were prescribed DOACs for the treatment of deep vein thrombosis. |

*Abbreviations: DOAC, Direct oral anticoagulant.*

**Supplemental Table 3**

**Patient characteristics and clinical outcome with and without albumin data of DIRECT-Extend study**

| **Variable** | **With Albumin** | **Without Albumin** | **P value** | **Missing (%)** |
| --- | --- | --- | --- | --- |
|  | **N = 2523** | **N = 4989** |  |  |
| Age | 73.0 [65.5, 80.0] | 72.0 [66.0, 78.0] | 0.001 | 0 |
| Female sex | 903 (35.8) | 1428 (28.6) | <0.001 | 0 |
| Body weight, Kg | 61.0 [52.0, 70.0] | 63.0 [54.0, 71.5] | <0.001 | 0.4 |
| Body mass index, Kg/m2 | 23.2 [20.5, 26.3] | 23.7 [21.3, 26.2] | <0.001 | 1.5 |
| AF type |  |  | <0.001 | 0 |
| Paroxysmal | 1523 (60.4) | 2230 (44.7) | |  |
| Persistent or long persistent | 917 (36.3) | 2697 (54.1) | |  |
| Unknown | 83 (3.3) | 62 (1.2) |  |  |
| Hypertension | 1674 (66.3) | 3518 (70.5) | <0.001 | 0 |
| Diabetes mellitus | 664 (26.3) | 1178 (23.6) | 0.011 | 0 |
| Dyslipidemia | 1258 (49.9) | 2293 (46.0) | 0.002 | 0 |
| Coronary artery disease | 346 (13.7) | 482 (9.7) | <0.001 | 0 |
| History of stroke | 674 (26.7) | 699 (14.0) | <0.001 | 0 |
| History of heart failure | 1001 (39.7) | 1072 (21.5) | <0.001 | 0 |
| History of bleeding | 422 (16.7) | 250 (5.0) | <0.001 | 0.1 |
| Vascular disease | 641 (25.4) | 748 (15.0) | <0.001 | 0 |
| Smoking |  |  | 0.024 | 1.3 |
| Never | 1235 (49.6) | 2375 (48.3) | |  |
| Past | 979 (39.3) | 1891 (38.4) | |  |
| Current | 276 (11.1) | 655 (13.3) | |  |
| Liver dysfunction | 591 (23.4) | 414 (23.6) | 0.911 | 43.1 |
| History of malignant neoplasm | 670 (26.6) | 256 (14.6) | <0.001 | 43.1 |
| CHADS_2_ score | 2.0 [1.0, 3.0] | 2.0 [1.0, 3.0] | <0.001 | 0 |
| CHA_2_DS_2_-VASc score | 4.0 [2.0, 5.0] | 3.0 [2.0, 4.0] | <0.001 | 0.1 |
| ORBIT score | 2.0 [1.0, 4.0] | 1.0 [0.0, 2.0] | <0.001 | 4.4 |
| DOAC score | 6.0 [4.0, 9.0] | 6.0 [3.0, 8.0] | <0.001 | 47.1 |
| HAS-BLED score | 3.0 [2.0, 4.0] | 3.00 [2.0, 4.0] | <0.001 | 46 |
| Major bleeding | 250 (9.9) | 312 (6.3) | <0.001 | 0 |
| Clinically relevant non-major bleeding | 1085 (43.0) | 1081 (21.7) | <0.001 | 0 |
| Any bleeding | 1148 (45.5) | 1240 (24.9) | <0.001 | 0 |
| Ischemic stroke | 62 (2.5) | 159 (3.2) | 0.090 | 0 |
| Systemic embolism | 48 (1.9) | 48 (1.0) | 0.001 | 0 |
| Hemorrhagic stroke | 35 (1.4) | 74 (1.5) | 0.821 | 0 |
| Heart failure admission | 256 (10.1) | 150 (8.6) | 0.092 | 43.1 |
| All cause death | 202 (8.0) | 281 (5.6) | <0.001 | 0 |

Data with listwise deletion are expressed as median [interquartile range] or number (percentage). Abbreviations: AF, Atrial fibrillation.

**Supplemental Table 4**

**Medications**

| **Variable** | **Lower tertile** | **Middle tertile** | **Higher tertile** | **P value** | **Missing** |
| --- | --- | --- | --- | --- | --- |
|  | **Alb < 3.7 g/dL** | **3.7 ≤ Alb <4.1 g/dL** | **Alb ≥ 4.1g/dL** |  |  |
|  | **N = 860** | **N = 835** | **N = 828** |  |  |
| DOAC type |  |  |  | 0.002 | 0 |
| Dabigatran | 134 (15.6) | 138 (16.5) | 164 (19.8) |  |  |
| Rivaroxaban | 212 (24.7) | 221 (26.5) | 237 (28.6) |  |  |
| Apixaban | 306 (35.6) | 276 (33.1) | 215 (26.0) |  |  |
| Edoxaban | 208 (24.2) | 200 (24.0) | 212 (25.6) |  |  |
| Low dose DOAC | 570 (66.3) | 465 (55.7) | 336 (40.6) | <0.001 | 0 |
| NSAIDs | 48 (5.6) | 26 (3.1) | 27 (3.3) | 0.014 | 0 |
| Antiplatelet therapy | 243 (28.3) | 200 (24.0) | 158 (19.1) | <0.001 | 0 |
| Aspirin | 197 (22.9) | 152 (18.2) | 121 (14.6) | <0.001 | 0 |
| Clopidogrel | 60 (7.0) | 50 (6.0) | 43 (5.2) | 0.306 | 0 |
| Ticlopidine | 1 (0.1) | 6 (0.7) | 0 (0.0) | 0.011 | 0 |
| Cilostazol | 21 (2.4) | 14 (1.7) | 14 (1.7) | 0.425 | 0 |
| Prasugrel | 16 (1.9) | 9 (1.1) | 13 (1.6) | 0.410 | 0 |
| Statin | 248 (28.8) | 270 (32.3) | 286 (34.5) | 0.040 | 0 |
| Proton pump inhibitor | 466 (54.2) | 394 (47.2) | 343 (41.4) | <0.001 | 0 |
| H_2_ blocker | 72 (8.4) | 56 (6.7) | 42 (5.1) | 0.026 | 0 |
| Pgp inhibitor | 124 (14.4) | 138 (16.5) | 121 (14.6) | 0.413 | 0 |

Data with listwise deletion are expressed as number (percentage).

Abbreviations: DOAC, Direct oral anticoagulant; NSAIDs, Non-steroidal anti-inflammatory drugs.

**Supplemental Table 5**

**Laboratory data**

| **Variable** | **Lower tertile** | **Middle tertile** | **Higher tertile** | **P value** | **Missing (%)** |
| --- | --- | --- | --- | --- | --- |
|  | **Alb < 3.7 g/dL** | **3.7 ≤ Alb <4.1 g/dL** | **Alb ≥ 4.1g/dL** |  |  |
|  | **N = 860** | **N = 835** | **N = 828** |  |  |
| Albumin, g/dL | 3.3 [3.0, 3.6] | 4.0 [3.9, 4.1] | 4.3 [4.2, 4.5] | <0.001 | 0 |
| Hemoglobin, g/dL | 11.7 [10.5, 13.1] | 13.3 [12.1, 14.5] | 14.3 [12.9, 15.3] | <0.001 | 0.1 |
| Platelets, 10^3/uL | 195.0 [149.8, 247.0] | 194.0 [157.0, 232.0] | 197.5 [166.0, 231.0] | 0.696 | 0.4 |
| Creatinine, mg/dL | 0.9 [0.7, 1.1] | 0.9 [0.7, 1.1] | 0.9 [0.7, 1.0] | 0.071 | 0.2 |
| Creatinine clearance, mL/min/1.73 m² | 54.6 [38.6, 75.4] | 59.5 [45.3, 77.1] | 71.84 [55.3, 90.3] | <0.001 | 0.3 |
| CRP, mg/L | 0.08 [0.04, 0.29] | 0.05 [0.04, 0.12] | 0.04 [0.04, 0.10] | <0.001 | 2.6 |
| BNP, pg/mL | 213.7 [96.3, 457.0] | 142.3 [66.1, 269.4] | 97.5 [40.0, 183.1] | <0.001 | 49 |
| NT-proBNP, pg/mL | 1722.0 [731.7, 3358.0] | 776.0 [326.0, 1832.0] | 509.0 [145.0, 1151.2] | <0.001 | 50.7 |
| Total Bilirubin, mg/dL | 0.6 [0.5, 0.9] | 0.7 [0.5, 0.9] | 0.7 [0.6, 1.0] | <0.001 | 3.4 |
| AST, IU/L | 25.0 [19.0, 35.0] | 23.0 [19.0, 30.0] | 25.0 [20.0, 32.0] | <0.001 | 0.3 |
| ALT, IU/L | 19.0 [13.0, 30.2] | 17.0 [13.0, 26.0] | 21.0 [15.0, 29.0] | <0.001 | 0.3 |

Data with listwise deletion are expressed as median [interquartile range].

Abbreviations: Alb, albumin; CRP, C-reactive protein; BNP, B-type natriuretic peptide; NT-proBNP, N-terminal pro b-type natriuretic peptide; AST, Aspartate aminotransferase; ALT, Alanine aminotransferase.

**Supplemental Table 6**

**Echocardiographic data**

| **Variable** | **Lower tertile** | **Middle tertile** | **Higher tertile** | **P value** | **Missing (%)** |
| --- | --- | --- | --- | --- | --- |
|  | **Alb < 3.7 g/dL** | **3.7 ≤ Alb <4.1 g/dL** | **Alb ≥ 4.1g/dL** |  |  |
|  | N = 860 | N = 835 | N = 828 |  |  |
| LVDd, mm | 48.0 [44.0, 53.0] | 48.0 [44.0, 52.0] | 49.0 [45.0, 54.0] | 0.008 | 32.6 |
| LVDs, mm | 31.0 [27.0, 38.0] | 31.0 [27.0, 36.0] | 31.0 [28.0, 37.0] | 0.180 | 32.7 |
| IVSTd, mm | 9.0 [8.0, 10.0] | 9.0 [8.0, 10.0] | 9.0 [8.0, 10.0] | 0.002 | 33 |
| LVPWTd, mm | 9.0 [8.0, 10.0] | 9.0 [8.0, 10.0] | 9.0 [8.0, 10.0] | 0.012 | 33 |
| LVEF, % | 63.0 [50.0, 70.0] | 64.0 [55.0, 70.0] | 64.0 [55.0, 70.0] | 0.173 | 33.1 |
| LADs, mm | 46.0 [40.2, 51.0] | 46.0 [41.0, 51.0] | 44.0 [40.0, 50.0] | 0.020 | 33.2 |
| TRPG, mmHg | 27.0 [22.0, 33.0] | 23.0 [19.0, 29.0] | 23.0 [19.0, 28.0] | < 0.001 | 46.4 |
| TAPSE, mm | 17.0 [13.5, 20.5] | 16.0 [12.5, 19.9] | 15.8 [12.9, 20.1] | 0.698 | 96 |

Data with listwise deletion are expressed as median [interquartile range].

Abbreviations: LVDd, left ventricular diastolic dimension; LVDs, left ventricular systolic dimension; IVSTd, interventricular septum thickness in diastole; LVPWTd, left ventricular posterior wall thickness in diastole; LVEF, left ventricular ejection fraction; LADs, left atrial dimension in systole; TRPG, tricuspid regurgitation pressure gradient; TAPSE, tricuspid annular plane systolic excursion.

**Supplemental Table 7**

**Impact of low dose DOAC on major bleeding in albumin tertiles**

| **Variable** | **Lower tertile** | | **Middle tertile** | | **Higher tertile** | |
| --- | --- | --- | --- | --- | --- | --- |
|  | **Alb < 3.7 g/dL** | | **3.7 ≤ Alb <4.1 g/dL** | | **Alb ≥ 4.1g/dL** | |
|  | **N = 860** |  | **N = 835** |  | **N = 828** |  |
|  | **HR [95% CI]** | **P value** | **HR [95% CI]** | **P value** | **HR [95% CI]** | **P value** |
| **Low dose DOAC** | 0.70 [0.44-1.12] | 0.137 | 0.92 [0.55-1.54] | 0.759 | 0.77 [0.41-1.43] | 0.402 |

Cox proportional hazards regression analysis for major bleeding was performed including several covariates such as age, female sex, body mass index, diabetes mellitus, hypertension, history of bleeding, history of stroke, malignant neoplasm, hemoglobin levels, creatinine clearance, C-reactive protein, liver dysfunction, antiplatelet therapy, and NSAIDs use.

Abbreviations: Alb, albumin; NSAIDs, Non-steroidal anti-inflammatory drugs; DOAC, Direct oral anticoagulant.

**Supplemental table 8**

**Patient characteristics stratified by DOACs**

| **Variable** | **Dabigatran** | **Rivaroxaban** | **Apixaban** | **Edoxaban** | **P value** | **Missing (%)** |
| --- | --- | --- | --- | --- | --- | --- |
|  | **N = 436** | **N = 670** | **N = 797** | **N = 620** |  |  |
| Age | 71.0 [64.0, 77.0] | 72.0 [63.0, 78.0] | 75.0 [67.0, 82.0] | 75.0 [67.0, 80.0] | <0.001 | 0 |
| Female | 116 (26.6) | 201 (30.0) | 321 (40.3) | 265 (42.7) | <0.001 | 0 |
| Body weight, Kg | 62.0 [54.2, 71.0] | 62.0 [53.8, 72.0] | 60.0 [50.6, 69.0] | 59.4 [51.0, 69.1] | <0.001 | 0.2 |
| Body mass index, Kg/m2 | 23.5 [20.8, 26.8] | 23.3 [20.6, 26.2] | 23.2 [20.1, 26.2] | 23.0 [20.5, 25.8] | 0.182 | 1.7 |
| AF type |  |  |  |  | 0.158 | 0 |
| Paroxysmal | 281 (64.4) | 402 (60.0) | 473 (59.3) | 367 (59.2) |  |  |
| Persistent or long persistent | 136 (31.2) | 243 (36.3) | 300 (37.6) | 238 (38.4) |  |  |
| Unknown | 19 (4.4) | 25 (3.7) | 24 (3.0) | 15 (2.4) |  |  |
| Hypertension | 286 (65.6) | 420 (62.7) | 558 (70.0) | 410 (66.1) | 0.030 | 0 |
| Diabetes mellitus | 115 (26.4) | 173 (25.8) | 231 (29.0) | 145 (23.4) | 0.124 | 0 |
| Dyslipidemia | 215 (49.3) | 309 (46.1) | 454 (57.0) | 280 (45.2) | <0.001 | 0 |
| Coronary artery disease | 56 (12.8) | 84 (12.5) | 124 (15.6) | 82 (13.2) | 0.324 | 0 |
| History of stroke | 129 (29.6) | 166 (24.8) | 241 (30.2) | 138 (22.3) | 0.002 | 0 |
| History of heart failure | 134 (30.7) | 248 (37.0) | 369 (46.3) | 250 (40.3) | <0.001 | 0 |
| History of bleeding | 67 (15.4) | 100 (14.9) | 163 (20.5) | 92 (14.8) | 0.009 | 0 |
| Vascular disease | 105 (24.1) | 156 (23.3) | 236 (29.6) | 144 (23.2) | 0.012 | 0 |
| Smoking |  |  |  |  | <0.001 | 1.3 |
| Never | 175 (40.7) | 312 (47.3) | 403 (51.1) | 345 (56.4) |  |  |
| Past | 187 (43.5) | 263 (39.9) | 314 (39.8) | 215 (35.1) |  |  |
| Current | 68 (15.8) | 84 (12.7) | 72 (9.1) | 52 (8.5) |  |  |
| Liver dysfunction | 110 (25.2) | 159 (23.7) | 196 (24.6) | 126 (20.3) | 0.188 | 0 |
| History of Malignant neoplasm | 95 (21.8) | 162 (24.2) | 239 (30.0) | 174 (28.1) | 0.006 | 0 |
| CHADS_2_ score | 2.0 [1.0, 3.0] | 2.0 [1.0, 3.0] | 2.0 [1.0, 4.0] | 2.0 [1.0, 3.0] | <0.001 | 0 |
| CHA_2_DS_2_-VASc | 3.0 [2.0, 5.0] | 3.0 [2.0, 5.0] | 4.0 [3.0, 5.0] | 4.0 [2.0, 5.0] | <0.001 | 0 |
| ORBIT score | 2.0 [0.0, 3.0] | 2.0 [0.0, 3.0] | 2.0 [1.0, 4.0] | 2.0 [1.0, 4.0] | <0.001 | 0.3 |
| DOAC score | 5.0 [3.0, 8.0] | 6.0 [3.0, 8.0] | 7.0 [5.0, 9.0] | 6.0 [4.0, 8.0] | <0.001 | 2 |
| HAS-BLED score | 3.0 [2.0, 4.0] | 3.0 [1.0, 4.0] | 3.0 [2.0, 4.0] | 3.0 [2.0, 4.0] | <0.001 | 0.2 |

Data with listwise deletion are expressed as median [interquartile range] or number (percentage).

Abbreviations: AF, Atrial fibrillation; DOAC, Direct oral anticoagulant

**Supplemental table 9**

**Medications stratified by DOACs**

| **Variable** | **Dabigatran** | **Rivaroxaban** | **Apixaban** | **Edoxaban** | **P value** | **Missing (%)** |
| --- | --- | --- | --- | --- | --- | --- |
|  | **N = 436** | **N = 670** | **N = 797** | **N = 620** |  |  |
| Low dose DOAC | 311 (71.3) | 257 (38.4) | 363 (45.5) | 440 (71.0) | <0.001 | 0 |
| Antiplatelet therapy | 104 (23.9) | 139 (20.7) | 224 (28.1) | 134 (21.6) | 0.004 | 0 |
| Aspirin | 91 (20.9) | 99 (14.8) | 175 (22.0) | 105 (16.9) | 0.002 | 0 |
| Clopidogrel | 29 (6.7) | 43 (6.4) | 51 (6.4) | 30 (4.8) | 0.531 | 0 |
| Ticlopidine | 3 (0.7) | 1 (0.1) | 2 (0.3) | 1 (0.2) | 0.337 | 0 |
| Cilostazol | 7 (1.6) | 15 (2.2) | 18 (2.3) | 9 (1.5) | 0.621 | 0 |
| Prasugrel | 2 (0.5) | 13 (1.9) | 12 (1.5) | 11 (1.8) | 0.224 | 0 |
| NSAIDs | 17 (3.9) | 27 (4.0) | 33 (4.1) | 24 (3.9) | 0.994 | 0 |
| Statin | 124 (28.4) | 195 (29.1) | 295 (37.0) | 190 (30.6) | 0.002 | 0 |
| Proton pump inhibitor | 196 (45.0) | 310 (46.3) | 412 (51.7) | 285 (46.0) | 0.052 | 0 |
| H_2_ blocker | 35 (8.0) | 51 (7.6) | 49 (6.1) | 35 (5.6) | 0.310 | 0 |
| Pgp inhibitor | 67 (15.4) | 107 (16.0) | 125 (15.7) | 84 (13.5) | 0.620 | 0 |

Data with listwise deletion are expressed as number (percentage).

Abbreviations: DOAC, Direct oral anticoagulant; NSAIDs, Non-steroidal anti-inflammatory drugs.

**Supplemental table 10**

**Laboratory data stratified by DOACs**

| **Variable** | **Dabigatran** | **Rivaroxaban** | **Apixaban** | **Edoxaban** | **P value** | **Missing (%)** |
| --- | --- | --- | --- | --- | --- | --- |
|  | **N = 436** | **N = 670** | **N = 797** | **N = 620** |  |  |
| Hemoglobin, g/dL | 13.5 [12.0, 14.8] | 13.3 [11.9, 14.8] | 12.9 [11.4, 14.3] | 12.9 [11.4, 14.5] | <0.001 | 0.1 |
| Platelets, 10^3/uL | 188.0 [149.0, 226.0] | 193.0 [158.0, 233.0] | 199.0 [157.0, 236.0] | 202.5 [164.0, 247.0] | 0.001 | 0.4 |
| Creatinine, mg/dL | 0.8 [0.7, 1.0] | 0.9 [0.7, 1.0] | 0.9 [0.7, 1.1] | 0.9 [0.7, 1.1] | <0.001 | 0.2 |
| CRP, mg/L | 0.1 [0.0, 0.2] | 0.1 [0.0, 0.1] | 0.1 [0.0, 0.2] | 0.0 [0.0, 0.1] | 0.206 | 2.6 |
| BNP, pg/mL | 114.2 [48.1, 250.0] | 121.0 [54.9, 265.6] | 154.4 [78.7, 335.6] | 143.6 [59.0, 296.6] | 0.007 | 49 |
| NT-proBNP, pg/mL | 1065.5  [406.6, 2178.8] | 711.4  [256.0, 1743.0] | 1052.0  [475.0, 2485.0] | 792.3  [293.9, 2132.5] | <0.001 | 50.7 |
| Albumin, g/dL | 4.0 [3.6, 4.3] | 4.0 [3.7, 4.3] | 3.9 [3.5, 4.2] | 4.0 [3.6, 4.3] | <0.001 | 0 |
| PT-INR | 1.1 [1.0, 1.3] | 1.2 [1.1, 1.5] | 1.1 [1.0, 1.3] | 1.2 [1.1, 1.5] | <0.001 | 16.7 |
| Total Bilirubin, mg/dL | 0.7 [0.5, 1.0] | 0.7 [0.5, 0.9] | 0.7 [0.5, 0.9] | 0.7 [0.5, 0.9] | 0.199 | 3.4 |
| AST, IU/L | 24.0 [19.0, 32.0] | 25.0 [20.0, 33.0] | 24.0 [20.0, 33.0] | 24.0 [19.0, 31.5] | 0.579 | 0.3 |
| ALT, IU/L | 21.0 [14.0, 30.0] | 19.0 [14.0, 30.0] | 19.0 [13.0, 28.0] | 18.0 [13.0, 26.0] | 0.002 | 0.3 |

Data with listwise deletion are expressed as median [interquartile range].

Abbreviations: DOAC, Direct oral anticoagulant; BNP, B-type natriuretic peptide; NT-proBNP, N-terminal pro b-type natriuretic peptide; AST, Aspartate aminotransferase; ALT, Alanine aminotransferase.

**Supplemental table 11**

**Pharmacokinetic characteristics of DOACs**

| **Characteristic** | **Dabigatran** | **Rivaroxaban** | **Apixaban** | **Edoxaban** |
| --- | --- | --- | --- | --- |
| Prodrug | Yes | No | No | No |
| Bioavailability | 3-7 (due to its high polarity) | 70 (without food) 100 (with food) | 50 | 62 |
| Time to maximum effect [Tmax (h)] | 1.5-2 h | 2-4 h | 1-3 h | 1-2 h |
| Volume of distribution [VD (L)] | 50-70 | 50 | 23 | 107 |
| Plasma protein binding | 35 | >90 | 87 | 55 |
| Half-life (h) | 12-14 | 5-9 (young adults) 11-13 (elderly) | ~12 | 10-14 |
| Metabolism | No (20% glucuronic acid conjugation) | (65%) CYP3A4, CYP2J2 | (73%) CYP3A4/5, 1A2, 2C8, 2C9, 2C19, 2J2 | (50%) CYP3A4/5 (<　10%) |
| Substrate for CYP3A4 | No | Yes | Yes | Yes |
| Substrate for P-gp | Yes, dabigatran etexilate | Yes | Yes | Yes |
| Substrate for other transporters | Unknown | BCRP | BCRP | Unknown |
| Elimination | 80% renal (unchanged) 20% liver | 33% renal 66% liver | 25% renal 75% liver | 50% renal 50% liver |
| Drug-drug interactions | P-gp | P-gp, CYP3A4 | P-gp, CYP3A4 | P-gp, CYP3A4 |
| Food-drug interactions | Prolongs Tmax to 2 h (Intake with food discouraged) | Mean AUC increases to ~40% (Intake with food mandatory) | No effect (Intake with food discouraged) | No effect (Intake with food: no official recommendation) |
| Daily doses required | Twice daily | Once daily | Twice daily | Once daily |

Abbreviations: DOAC, Direct oral anticoagulant; VD, Volume of distribution; Tmax, Time to maximum effect; CYP3A4, Cytochrome P450 3A4; P-gp, P-glycoprotein; BCRP, Breast cancer resistance protein; AUC, Area under the curve.
